# *Ewingella americana* Infections in Humans—A Narrative Review

**DOI:** 10.3390/antibiotics13060559

**Published:** 2024-06-14

**Authors:** Petros Ioannou, Stella Baliou, Diamantis Kofteridis

**Affiliations:** School of Medicine, University of Crete, 71003 Heraklion, Greece

**Keywords:** bacteremia, *Ewingella americana*, peritoneal dialysis peritonitis

## Abstract

*Ewingella americana* is a Gram-negative rod that belongs to the order Enterobacterales and the family Yersiniaceae and was first identified in 1983 from 10 clinical strains in the United States of America. The present study aimed to identify all the published cases of *E. americana* in the literature, describe the epidemiological, clinical, and microbiological characteristics, and provide data regarding its antimicrobial resistance, treatment, and outcomes. A narrative review was performed based on a PubMed and Scopus databases search. In total, 16 studies provided data on 19 patients with infections by *E. americana*. The median age of the patients was 55 years, and 47.4% were male. The most common infections were those of the bloodstream, the respiratory tract, and the peritoneal cavity. Antimicrobial resistance to cephalosporins, aminoglycosides, and the combination of trimethoprim with sulfamethoxazole was minimal, and these were the most commonly used antimicrobials for treating these infections. No included study provided information on the genetic or molecular mechanism of this pathogen’s antimicrobial resistance. The overall mortality was minimal, with only one patient with bacteremia succumbing to the infection. Further studies are needed to better understand this microorganism, its pathogenic potential in humans, and the genetic and molecular mechanisms underlying its antimicrobial resistance, for which very little evidence exists to date.

## 1. Introduction

The latest genetic and molecular tools that have been introduced in everyday practice in large microbiological laboratories, such as 16s rRNA and matrix-assisted laser desorption/ionization time-of-flight mass spectrometry (MALDI-TOF MS), have led to more frequent identification of novel microorganisms [1,2]. This is associated with the higher accuracy of these techniques to accurately identify microorganisms that are difficult to identify by classic microbiological means of morphology and biochemical profiles [3].

*Ewingella americana* is a Gram-negative rod that belongs to the order Enterobacterales and the family Yersiniaceae. This microorganism was first identified by Grimont et al. in 1983. In that study, Grimont et al. used ten clinical strains identified in the United States of America and described their morphologic, biochemical, and genetic characteristics in detail [4]. *E. americana* is rod-shaped, non-fluorescent, catalase-positive, oxidase-negative, lactose-fermenting, motile, and facultatively anaerobic [5,6]. This bacterium can be found in many different hosts, such as humans [7], plants [8,9], mushrooms [10], mollusks [11], and vacuum-packed meat [12]. To date, a small number of these species’ genomes are known and are publicly available in public genome databases such as the National Centre for Biotechnology Information (NCBI) genome database [6]. However, little research has been conducted on comprehensively analyzing this microorganism’s genome. In a recent study, Liu et al. used Single-Molecule-Real-Time (SMRT) technology to study the whole genome of *E. americana* B6-1 that had been isolated from cultivated *F. filiformis* [6]. That study revealed that these strains expressed common genes for virulence effectors, type secretion, carbohydrate-active enzymes, and toxins associated with pathogenicity in hosts. They also encoded genes associated with antimicrobial resistance, pigments for the evasion of host defenses, and genes associated with adaptation to different environmental settings, such as the presence or absence of specific nutrients, different temperatures, and oxidation [6]. Even though pathogenicity studies have not been conducted to draw safe conclusions, several virulence genes were found in the genomes of *E. americana* strains in that particular study. The study of genes associated with pathogenicity implies that *E. americana* may be an opportunistic pathogen that may primarily infect immunosuppressed patients [6].

This microorganism has rarely been identified as a cause of infection. Thus, its pathogenic potential has not been adequately described. For example, even though there are reports of infection by this pathogen [13,14], there are also reports of the identification of the microorganism in the context of colonization or pseudo-outbreaks [15,16].

Moreover, in cases of true infections by this microorganism, the epidemiological, clinical, and microbiological characteristics have not been adequately summarized in the literature. The present study aimed to identify all the published cases of *E. americana* in the literature, describe the epidemiological, clinical, and microbiological characteristics, and provide data regarding its antimicrobial resistance, treatment, and outcomes.

## 2. Results

### 2.1. Included Studies’ Characteristics

**Figure 1:**
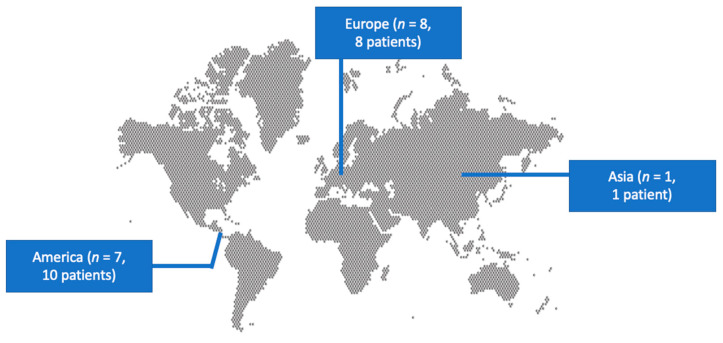
Geographical distribution of *Ewingella americana* human infections worldwide.

The literature search of the PubMed and Scopus databases yielded 72 non-duplicate studies. After screening all the potentially eligible articles and assessing the references of the included articles, only 16 studies met the inclusion criteria and were considered for data extraction [7,13,14,17,18,19,20,21,22,23,24,25,26,27,28,29]. These 16 studies provided information on 19 patients. Among them, eight studies were conducted in Europe, seven in North and South America, and one in Asia. There were 15 case reports and 1 case series. Figure 1 shows a graphical representation of the geographical distribution of the published cases. Table 1 shows the characteristics of the studies included in the present review.

### 2.2. Epidemiology of E. americana Infections

The patients with an infection by *E. americana* had an age range from 0 to 77 years [7,13,14,17,18,19,20,21,22,23,24,25,26,27,28,29]. The median was 55 years. Out of 19 patients, 9 (47.4%) were male [7,13,14,17,18,19,20,21,22,23,24,25,26,27,28,29]. Regarding the medical history, 31.6% (6 patients) had surgery in the three months before the diagnosis of infection by *E. americana*, with 15.8% (3 patients) having had cardiac surgery [13,14,18]. Among all the patients, 26.3% (5 patients) had recent antimicrobial use (within three months before diagnosis of the infection) [14,18], 15.8% (3 patients) had a history of end-stage renal disease (ESRD) and were on peritoneal dialysis [19,26,28], 5.3% (1 patient) had ESRD and was on hemodialysis [29], 15.8% (3 patients) had immunosuppression and, more specifically, 5.3% (1 patient) had an autoimmune syndrome (inflammatory bowel disease) and was on immunosuppression, 10.5% (2 patients) had organ transplantation, with 5.3% (1 patient) having kidney transplantation and 5.3% (1 patient) having allogenous bone marrow transplantation [20,22,23]. Additionally, 5.3% (1 patient) was an intravenous drug user [24].

### 2.3. Microbiology and Antimicrobial Resistance of E. americana Infections

*E. americana* was isolated from the blood in 47.4% (9 patients) [13,14,18,20,21,25], the peritoneal fluid in 15.8% (3 patients) [19,26,28], the sputum in 10.5% (2 patients), a swab from the conjunctiva in 10.5% (2 patients) [7,17], the synovial fluid in 5.3% (1 patient) [24], the urine in 5.3% (1 patient) [29], and the cerebrospinal fluid in 5.3% (1 patient) [27]. Infection was polymicrobial in 10.5% (2 patients), and the other identified species were *Pseudomonas marginata* and *Enterococcus faecalis* [13,29]. Identification was based on biochemical characteristics in 31.6% (6 patients), on VITEK (Biomerieux, Marcy-l'Étoile, France) in 21.1% (4 patients), on API 20 in 10.5% (2 patients), on MALDI-TOF MS in 10.5% (2 patients), on BBLCrystal Enteric/Nonfermenter ID Kit in 5.3% (1 patient), and in 16s rRNA sequencing in 5.3% (1 patient) [7,13,14,17,18,19,20,22,25,27,28]. The identification method was not reported in 26.3% (5 patients) [21,23,24,26,29].

The most commonly used method for susceptibility testing was disk diffusion in 78.6% (11 out of 14 patients with available data), E-test in 14.3% (2 patients), and microdilution in 7.1% (1 patient) [7,13,14,17,18,19,20,22,23,24,25]. Resistance to quinolones was found in 15.4% (2 out of 13 patients) [7,17,18,19,20,22,23,24,25,26,27,28,29], aminopenicillin resistance was observed in 11.8% (2 out of 17 patients) [7,13,14,17,18,19,20,22,23,24,25,26,27,28], resistance to tetracyclines was observed in 9.1% (1 out of 11 patients) [7,13,14,22,23,25,27,29], resistance to carbapenems was observed in 8.3% (1 out of 12 patients) [7,14,18,22,23,25,26,27,29], resistance to trimethoprim and sulfamethoxazole was observed in 6.7% (1 out of 15 patients) [7,13,14,17,18,22,23,24,25,26,27,28,29], and resistance to cephalosporins was observed in 5.6% (1 out of 18 patients) [7,13,14,17,18,19,20,22,23,24,25,26,27,28,29].

### 2.4. Clinical Presentation of E. americana Infections

The most common *E. americana* infections were those of the bloodstream in 47.4% (9 out of 19 patients) [13,14,18,20,21,25], peritonitis in 15.8% (3 patients) [19,26,28], lower respiratory tract infections in 15.8% (3 patients) [22,23,25], conjunctivitis in 10.5% (2 patients) [7,17], central nervous system infections in 5.3% (1 patient) [27], urinary tract infection in 5.3% (1 patient) [29], and bone and joint infection in 5.3% (1 patient) [24]. The most common clinical symptoms were fever in 57.9% (11 patients) and sepsis in 53.3% (8 out of 15 patients) [7,13,14,17,18,19,20,21,22,23,24,25,26,27,28,29].

### 2.5. Treatment and Outcomes of E. americana Infections

The antimicrobials that were more commonly used were aminoglycosides in 47.4% (9 out of 19 patients), trimethoprim and sulfamethoxazole in 26.3% (5 patients), cephalosporins in 26.3% (5 patients), aminopenicillin with a beta-lactamase inhibitor in 15.8% (3 patients), mezlocillin in 10.5% (2 patients), carbapenem in 5.3% (1 patient), carbenicillin in 5.3% (1 patient), temocillin in 5.3% (1 patient), aminopenicillin in 5.3% (1 patient), tetracycline in 5.3% (1 patient), and colistin in 5.3% (1 patient) [7,13,14,17,18,19,20,21,22,23,24,25,26,27,28,29]. The median treatment duration among the survivors was 15.5 days, the range was 10 to 42 days, and the interquartile range was 10 to 21 days [22,23,24,25,26,27,28,29]. The overall mortality was 5.3% (1 out of 19 patients) and was attributed directly to the infection [7,13,14,17,18,19,20,21,22,23,24,25,26,27,28,29]. Table 2 shows the characteristics of patients with infection by *E. americana* in total and in regard to the type of infection.

### 2.6. Bacteremia Due to E. americana

Nine patients had bacteremia by *E. americana*. Their median age was 57 years, with a range of 4 to 75 years and an interquartile range of 47.5 to 66 years; 44.4% (4 out of 9 patients) were male [7,13,14,17,18,19,20,21,22,23,24,25,26,27,28,29]. Among all the patients, 66.7% (6 patients) had recent surgery, more commonly cardiac surgery in 33.3% (3 patients) [13,14], and 11.1% (1 patient) had immunosuppression due to hematologic malignancy that had been treated with allogenous bone marrow transplantation [20]. Moreover, 40% (2 out of 5 patients with available data) had a central venous catheter [13,14,18,20,21,25], and 55.6% (5 out of 9 patients) had recently received antimicrobials [13,14,18,20,21,25]. The infection was polymicrobial in 11.1% (1 patient) and was community-acquired only in 22.2% (2 patients), with the remaining 77.8% (7 patients) having a hospital-acquired infection [7,13,14,17,18,19,20,21,22,23,24,25,26,27,28,29]. Fever was noted in 88.9% (8 patients) [7,13,14,17,18,19,20,21,22,23,24,25,26,27,28,29] and sepsis in 100% (5 out of 5 patients with available data) [13,14,18,20,21,25]. Mortality was 11.1% (1 out of 9 patients) and was directly attributed to the infection [13,14,18,20,21,25].

### 2.7. Respiratory Tract Infection Due to E. americana

Three patients had respiratory tract infections by *E. americana* [22,23,25]. Their median age was 35 years, ranging from 4 to 77 years; 33.3% (1 out of 3 patients) were male [22,23,25]. Among all the patients, 66.7% (2 patients) had immunosuppression, one due to an autoimmune syndrome (inflammatory bowel disease treated with mercaptopurine) and one due to kidney transplantation that was treated with immunosuppression in both cases [22,23]. In 33.3% (1 patient), a diagnosis of bloodstream infection was also made [25]. The infection was community-acquired in 66.7% (2 patients) and hospital-acquired in 33.3% (1 patient) [22,23,25]. Fever was present in 66.7% (2 patients), and sepsis was also diagnosed in 66.7% (2 patients) [22,23,25]. The median duration of treatment was 10 days. No patient died from the infection [22,23,25].

### 2.8. Peritonitis Due to E. americana

In the present review, three patients had peritonitis due to *E. americana* [19,26,28]. The median age of these patients was 68 years, ranging from 45 to 70 years; none were male [19,26,28]. All the patients had ESRD and were on peritoneal dialysis (PD) [19,26,28]. The diagnosis was based on analyzing the peritoneal fluid through white cell numbers and cultures. No patient had fever or sepsis, and the presenting clinical symptom was abdominal pain [19,26,28]. The median duration of the treatment was 21 days and was provided intraperitoneally in all the patients. No patient died or had to be transitioned to hemodialysis after an episode of PD-associated peritonitis [19,26,28].

### 2.9. Conjunctivitis Due to E. americana

Herein, two patients had conjunctivitis by *E. americana* [7,17]; one was male [7], and one was female [17], while their ages were 3 and 30 years, respectively. No predisposing factors were noted in the patients’ medical histories [7,17]. Infection was community-acquired in both cases [7,17]. The symptoms were only local in both cases. Local treatment was provided in both patients; however, in both cases, oral treatment had to be provided to resolve the infection symptoms. The outcome was optimal in both cases, with no sequelae regarding their vision [7,17].

### 2.10. Other Infections Due to E. americana

Three more patients had infections by *E. americana*; one 73-year-old male had a urinary tract infection [29], one 50-year-old male intravenous drug user had a bone and joint infection [24], and one male neonate had a central nervous system infection [27]. All the other patients presented with fever and sepsis except for the bone and joint infection patient. The outcome was optimal in all the cases [24,27,29].

## 3. Discussion

The present study summarizes the characteristics of the patients diagnosed with an infection by *E. americana* based on previously published studies and provides information regarding the epidemiology, microbiology, clinical characteristics, treatment, and mortality. The most common types of infection were those of the bloodstream, PD-associated peritonitis, respiratory tract infection, and conjunctivitis. The most commonly used antimicrobials for the treatment of these infections were aminoglycosides, a combination of trimethoprim and sulfamethoxazole, and cephalosporins. Overall, the mortality was very low.

*E. americana* was recently characterized as a separate species and is rarely identified as a cause of infection, so the exact epidemiology, microbiology, and clinical characteristics have yet to be adequately described. In this review, most patients were female, and the median age was 55 years. Most studies were conducted in North America and Europe. The geographic distribution of the patients in the present review may be closely associated with the need for advanced diagnostic modalities, such as genetic testing and MALDI-TOF MS, to identify the microorganisms. Thus, the geographic distribution shown herein may not fully represent the true epidemiology of the pathogen since cases of the pathogen in areas where such diagnostic modalities are not available could be misdiagnosed. Since this microorganism can be found in many different hosts, such as humans and plants [8,9,10], considerations regarding the transmission of this microorganism to different hosts would be plausible. Thus, the possibility of *E. americana* infections being zoonotic should be further evaluated. However, the type of infection in most patients in this review was not community-acquired and was health-care-associated or hospital-acquired. This could imply that the pathogen could be a colonizer of the hospital environment. In 1987, McNeil et al. described a series of 20 patients initially considered to have had bacteremia by *E. americana* in a hospital in the USA. However, after careful analysis, this proved to be a pseudo-epidemic that was associated with contaminated coagulation tubes and a non-sterile method of blood collection for blood cultures by phlebotomists [16]. Even though bacteremia was not true in that study, colonization of the hospital environment by this pathogen was proved. Patients exposed to a hospital environment that these bacteria have colonized are at risk for colonization and infection by the same microorganisms [30]. Pathogens can survive for a prolonged time in hospital environments, and hospital-acquired infections can occur in the context of patient exposure to healthcare workers, surfaces, or other patients’ colonized items. This risk could be reduced through adequate cleaning and infection control practices, such as thorough hand hygiene practices [30,31,32,33,34,35]. Of note, a significant proportion of patients who had bacteremia were recovering from surgery [13,14,18]. This could imply a closer association with the healthcare environment since half of those patients who were recovering from surgery had cardiac surgery and others had vascular surgery [14]. These major surgical procedures often require a post-surgical stay in the intensive care unit and instrumentation that increases the risk of hospital-acquired infections [36,37]. Thus, the zoonotic potential of this pathogen could not be identified in the present review. However, further studies could formally evaluate this possibility.

Another frequent condition in the medical histories of patients with infection by *E. americana* was immunosuppression. More specifically, three patients had immunosuppression due to pharmacologic treatment of an autoimmune condition, kidney transplantation, and allogenous bone marrow transplantation [20,22,23]. Indeed, immunosuppression is associated with an increased risk of infection [38]. However, the defect in the immune response defines the type of susceptibility to infection. Thus, patients with primary immunodeficiencies such as chronic granulomatous disease and severe combined immunodeficiency may both have an increased likelihood of infections from an early age since they are both primary immunodeficiencies. Still, due to the different mechanisms underlying the immunosuppression, the clinical presentation and the occurring infections differ [39,40]. In the studies describing *E. americana* infections in patients with immunosuppression in the present review, all the patients had acquired immunosuppression due to pharmacological treatment of autoimmune disorders or after organ transplantation. Mercaptopurine and azathioprine have been widely used in the treatment of inflammatory bowel disease; however, the risk of infection by opportunistic pathogens is increased [41,42]. For example, an increased risk of viral, mycobacterial, and fungal infection has been described with these drugs [42]. The bacterial infection risk has also been shown to be notable yet lower with these immunosuppressive medications [43]. Infections are a leading cause of mortality in patients who are solid organ transplantation recipients, with differences depending on the organ that has been transplanted. The infection risk in solid organ transplant recipients can be related to the organ that has been transplanted and the immunosuppressive treatment [44,45]. For example, patients with kidney transplantation are at an increased risk for urinary tract infections, especially for the first six months after kidney transplantation [46]. Similarly, patients with lung transplantation are at an increased risk of invasive fungal infection, especially invasive aspergillosis [47]. Bacterial infections are among the more frequent infections in such patients, including bacteremia, respiratory tract infections, and other infections. In the present study, two patients had a respiratory tract infection, and one had bacteremia. A recent study by Eichenberger et al. evaluated the differences in patients with and without solid organ transplantation and identified a similar mortality rate among these two different patient groups that could be associated with an earlier identification of the symptoms and time to appropriate treatment and the less pronounced immune response that could reduce the likelihood for an aberrant immune response that could lead to severe sepsis and shock [48,49]. Similarly, patients with bone marrow transplantation or hematopoietic stem cell transplantation are also at increased risk for bacterial, viral, and fungal infections; however, the increased incidence of these infections was not associated with a proportionally increased risk of mortality [50].

Identifying *E. americana* could be difficult since most microbiology laboratories do not have advanced molecular methods such as MALDI-TOF or gene sequencing, which are frequently needed for definite identification of infrequent bacteria [51]. In the studies included in the present study, genetic techniques such as 16s rRNA sequencing and MALDI-TOF MS led to accurate identification in all the cases. However, in most studies, identification was possible even with phenotypic and biochemical characteristics [7,13,14,17,18,19,22]. In many cases, automated identification methods, such as API and Vitek, aided this [7,18,19,22]. This is important since genetic testing and MALDI-TOF MS are not readily available in most laboratories.

There is no official guidance regarding the treatment of infections by *E. americana*. This pathogen is an infrequent cause of infections and may be underdiagnosed. Thus, its antimicrobial resistance patterns are of great interest. Most studies in the present review provided information regarding its antimicrobial susceptibility. Based on the available data from the included studies, resistance to aminopenicillins with or without a beta-lactamase inhibitor was significant, but resistance to cephalosporins and a combination of trimethoprim and sulfamethoxazole was minimal, and resistance to aminoglycosides was zero. Even though the mechanisms of antimicrobial resistance have not yet been fully elucidated, some studies are addressing this issue. For example, Liu et al., in 2020, performed a comparative genomic analysis by sequencing a high-quality genome of *E. americana* isolated from mushrooms and comparing it to four genomes of the same microorganism that was isolated from other origins [6]. In the same study, the authors evaluated the antimicrobial susceptibility of *E. americana.* Based on the complete antibiotic resistance (CARD) database, 27 unique antibiotic resistance genes were revealed in all the *E. americana* strain genomes and four distinct resistance pathways. Antibiotic resistance genes (ranging from 20 to 33) in the strains were linked to resistance to several antimicrobials, such as aminoglycoside, carbapenem, cephalosporin, fluoroquinolone, fosfomycin, macrolide, nitroimidazole, and tetracycline. The fosfomycin resistance gene (*fosA*) was discovered in all the strains. These strains’ most common antimicrobial resistance gene families encode multi-efflux pumps (23 genes). Notably, the present review suggests that *E. americana* may be hospital-acquired in most cases. This is an important parameter for a clinician in terms of antimicrobial resistance since the local antimicrobial prescription practices and the local antimicrobial resistance patterns in other bacteria may be of particular importance when considering the potential for antimicrobial resistance of hospital-acquired *E. americana* infections. This is because resistance can occur in the hospital either by selection of resistant microorganisms after exposure to antimicrobials or by transfer of genes providing resistance from other resistant microorganisms [52,53,54].

Hence, it is reasonable that based on the studies included in the present review, the most frequently used antimicrobials were aminoglycosides, cephalosporins, and a combination of trimethoprim with sulfamethoxazole.

This review had some notable limitations, mainly related to its narrative design. First, the literature search may have been incomplete, and some studies may have been missed due to the search strategy, even if a thorough search methodology was implemented. Second, due to the rarity of these infections, the present study included only case reports and case series. Finally, some information was missing in the included studies; thus, this study only presents and analyzes the available data from the included reports. Importantly, none of the included studies provided information on the genetic and molecular mechanisms conferring antimicrobial resistance to particular antimicrobials.

## 4. Materials and Methods

The methodology used in the present narrative review included screening the literature to identify original studies providing information on infections of humans by *E. americana*. More specifically, two investigators (P.I. and S.B.) independently searched the PubMed and Scopus databases for eligible articles reporting “*Ewingella* AND *americana* AND infection” until 25 March 2024. Any differences between the decisions regarding study inclusion were solved by consensus among the two investigators. Herein, only original information regarding human infections was included. Thus, reports of infections from case reports and case series providing data at least about epidemiology, microbiology, treatment, and the outcomes of *E. americana* infections in humans were included. Reviews, systematic reviews, letters to the editor, and any other non-original studies were excluded. Studies written in languages other than English, those with no access to the full text, studies presenting aggregated data, and those referring to animals were also excluded. Moreover, studies without information on patients’ epidemiology and mortality were excluded. The references of the included articles were searched to assess other potential studies that may have been missed during the screening procedure.

Two investigators (P.I. and S.B.) used a pre-defined template to extract all the relevant information from the eligible studies. Data regarding age, epidemiology characteristics, infection site, microbiology, antimicrobial susceptibility, antimicrobial treatment, and the outcomes of *E. americana* infections in humans were extracted and further analyzed for this review.

## 5. Conclusions

This study provides essential data about the epidemiology, clinical characteristics, microbiology, antimicrobial susceptibility, treatment, and outcomes of *E. americana* infections. The most common infections were those of the bloodstream, the respiratory tract, and the peritoneal cavity. In patients with bacteremia, surgery within the previous three months was very common, while peritonitis only occurred in patients undergoing peritoneal dialysis. Susceptibility to aminoglycosides, cephalosporins, and a combination of trimethoprim and sulfamethoxazole was very high, and these were the most commonly used antimicrobials for treating these infections. The infection outcome was optimal in almost all the cases. Further studies are needed to better understand this microorganism, its pathogenic potential in humans, and the genetic and molecular mechanisms underlying its antimicrobial resistance, for which little evidence exists to date.

## Figures and Tables

**Table 1:** Characteristics of included studies reporting *Ewingella americana* human infections.

Author, Year	Number of Patients	Gender	Age (Years)	Type of Infection	Treatment (%)	Mortality (%)
Pien et al., 1983 [13]	1	Male	41	Bacteremia	Aminoglycoside Trimethoprim and sulfamethoxazole	0 (0)
Pien et al., 1986 [14]	4	2 males 2 females	54, 55, 57, 58	4 patients with bacteremia	Aminoglycoside 4 (100) Trimethoprim and sulfamethoxazole 2 (50) Cephalosporin 1 (25) Aminopenicillin 1 (25) Carbenicillin 1 (25) Mezlocillin 2 (50) Tetracycline 1 (25)	0 (0)
Heizmann et al., 1991 [17]	1	Female	30	Conjunctivitis	Trimethoprim and sulfamethoxazole Colistin locally Aminoglycoside locally Aminopenicillin and beta-lactamase inhibitor	0 (0)
Devreese et al., 1992 [18]	1	Male	75	Bacteremia	Temocillin	0 (0)
Kati et al., 1999 [19]	1	Female	70	Peritoneal dialysis peritonitis	Aminoglycoside	0 (0)
Maertens et al., 2001 [20]	1	Female	57	Bacteremia	No antibiotics	0 (0)
Tsokos et al., 2003 [21]	1	Female	74	Bacteremia	No antibiotics	1 (100)
Ryoo et al., 2005 [22]	1	Male	35	Lower respiratory tract infection	Aminoglycoside Cephalosporin	0 (0)
Pound et al., 2007 [23]	1	Female	77	Lower respiratory tract infection	Trimethoprim and sulfamethoxazole	0 (0)
Hassan et al., 2012 [24]	1	Male	50	Bone and joint infection	Cephalosporin	0 (0)
Maraki et al., 2012 [7]	1	Male	3	Conjunctivitis	Aminopenicillin and beta-lactamase inhibitor	0 (0)
Esposito et al., 2019 [25]	1	Female	4	Lower respiratory tract infection and bacteremia	Macrolide Aminopenicillin and beta-lactamase inhibitor	0 (0)
Khurana et al., 2020 [26]	1	Female	68	Peritoneal dialysis peritonitis	Aminoglycoside	0 (0)
Meisler et al., 2020 [27]	1	Male	0	CNS infection	Cephalosporin	0 (0)
Abrantes et al., 2022 [28]	1	Female	45	Peritoneal dialysis peritonitis	Cephalosporin	0 (0)
Hourizadeh et al., 2023 [29]	1	Male	73	Urinary tract infection	Carbapenem	0 (0)

CNS: central nervous system.

**Table 2:** Characteristics of the different types of infections by *Ewingella americana*.

Characteristic *	All Patients (*n* = 19)	Bacteremia ** (*n* = 9)	Respiratory Tract Infection ** (*n* = 3)	PD-Associated Peritonitis (*n* = 3)	Conjunctivitis (*n* = 2)	Urinary Tract Infection (*n* = 1)	Bone and Joint Infection (*n* = 1)	Central Nervous System Infection (*n* = 1)
Age, median in years (IQR)	55 (35–70)	57 (47.5–66)	35 (4–77)	68 (45–70)	16.5 (3–30)	73	50	0
Male, *n* (%)	9 (47.4)	4 (44.4)	1 (33.3)	0 (0)	1 (50)	1 (100)	1 (100)	1 (100)
Post-surgery (within 3 months), *n* (%)	6 (31.6)	6 (66.7)	0 (0)	0 (0)	0 (0)	0 (0)	0 (0)	0 (0)
Post-cardiac surgery (within 3 months), *n* (%)	3 (15.8)	3 (33.3)	0 (0)	0 (0)	0 (0)	0 (0)	0 (0)	0 (0)
Previous antimicrobial therapy, *n* (%)	5 (26.3)	5 (55.6)	0 (0)	0 (0)	0 (0)	0 (0)	0 (0)	0 (0)
IVDU, *n* (%)	1 (5.3)	0 (0)	0 (0)	0 (0)	0 (0)	0 (0)	1 (100)	0 (0)
Immunosuppression, *n* (%)	3 (15.8)	1 (11.1)	2 (66.7)	0 (0)	0 (0)	0 (0)	0 (0)	0 (0)
ESRD on PD, *n* (%)	3 (15.8)	0 (0)	0 (0)	3 (100)	0 (0)	0 (0)	0 (0)	0 (0)
ESRD on HD, *n* (%)	1 (5.3)	0 (0)	0 (0)	0 (0)	0 (0)	1 (100)	0 (0)	0 (0)
Polymicrobial infection, *n* (%)	2 (10.5)	1 (11.1)	0 (0)	0 (0)	0 (0)	1 (100)	0 (0)	0 (0)
Community-acquired, *n* (%)	6 (31.6)	2 (22.2)	2 (66.7)	0 (0)	2 (100)	0 (0)	1 (100)	0 (0)
Clinical characteristics								
Fever, *n* (%)	11 (57.9)	8 (88.9)	2 (66.7)	0 (0)	0 (0)	1 (100)	0 (0)	1 (100)
Sepsis, *n* (%)	8/15 (53.3)	5/5 (100)	2 (66.7)	0 (0)	0 (0)	1 (100)	0 (0)	1 (100)
Outcomes								
Overall mortality, *n* (%)	1 (5.3)	1 (11.1)	0 (0)	0 (0)	0 (0)	0 (0)	0 (0)	0 (0)
Infection-related mortality, *n* (%)	1 (5.3)	1 (11.1)	0 (0)	0 (0)	0 (0)	0 (0)	0 (0)	0 (0)

ESRD: end-stage renal disease; HD: hemodialysis; IQR: interquartile range; IVDU: intravenous drug use; PD: peritoneal dialysis; * denominator is the total number of patients except if otherwise mentioned, ** cases of bacteremia include one case of respiratory tract infection with bacteremia.

## Data Availability

The data presented in this study are available upon request from the corresponding author.
